# Complementary and Alternative Therapies for Pediatric Attention Deficit Hyperactivity Disorder: A Descriptive Review

**DOI:** 10.5402/2012/804127

**Published:** 2012-12-26

**Authors:** H. Russell Searight, Kayla Robertson, Todd Smith, Scott Perkins, Barbara K. Searight

**Affiliations:** ^1^Department of Psychology, Lake Superior State University, 650 West Easterday Avenue, Sault Sainte Marie, MI 49783, USA; ^2^School of Social Work, University of Illinois, Urbana, IL 61801, USA; ^3^Department of Psychology, St. Catharine College, St. Catharine, Catharine, KY 40061, USA; ^4^Department of Psychology, Western Kentucky University, Bowling Green, KY 42101-3576, USA; ^5^Algoma University Sault Marie, ON, Canada P6A 2G4

## Abstract

Attention deficit hyperactivity disorder (AD/HD), characterized by impulsivity, distractibility, and inattention, has an estimated pediatric population prevalence of 6–8%. Family physicians and pediatricians evaluate and treat the majority of children with this condition. The evidence-based treatment of choice for ADHD, stimulant medication, continues to be a source of public controversy. Surveys suggest that among parents of children with ADHD, there is considerable interest in complementary and alternative medicine (CAM). These therapies include herbal preparations, mineral supplements, sugar restriction, and polyunsaturated fatty acids. Other AD/HD therapies include neuro-feedback, cognitive training, mindfulness meditation, and exposure to “green space.” In order to assist physicians and mental health professionals in responding to patient and parent queries, this paper briefly describes these CAM therapies and current research regarding their effectiveness. While investigations in this area are hampered by research design issues such as sample size and the absence of double-blind placebo-controlled trials, there is some evidence that omega three fatty acids, zinc supplements, and neuro-feedback may have some efficacy.

## 1. Introduction 

Attention deficit hyperactivity disorder (AD/HD) is a psychiatric condition affecting an estimated 5–7% of children [1]. AD/HD's core symptoms of elevated impulsivity, increased motor activity, impaired concentration, and short-term memory deficits are often chronic with their symptomatic expression changing with development [2]. While stimulant pharmacotherapy is the evidence-based treatment of choice for AD/HD [3], complementary and alternative (CAM) therapies are becoming increasingly common treatments for the condition.

In addition to chronicity, AD/HD has several other characteristics that make it a focus for CAM. While AD/HD is generally believed to stem from neurophysiological deficits [3], a precise etiology has yet to be established. The treatment of choice for the condition is a Schedule II stimulant medication with addiction potential. Stimulant medication, while efficacious, may be associated with side effects including facial tics, hypertension, and anorexia [3]. Finally, an estimated 20–30% of children with AD/HD do not respond to stimulant medication [4].

Surveys suggest that up to 60% of US patients use complementary and alternative medicine—with half of this group using CAM in the past year [5]. Children with chronic conditions are far more likely than the general pediatric population to use CAM [6]. In an Australian survey, 64% of parents of children with AD/HD reported using some form of CAM [7].

As a result of these factors, physicians diagnosing and treating ADHD are likely to be asked about the types, availability, and effectiveness of alternative therapies. CAM therapies vary widely and include homeopathy, dietary restriction and supplements, herbal products, biofeedback, and attention training. While randomized placebo-controlled trials (RCTs) are the standard for judging therapeutic efficacy, few of the CAM therapies have been subjected to this level of rigor [8]. This paper's purpose is to familiarize mental health professionals and primary care clinicians with common complementary and alternative AD/HD therapies as well as the current evidence of their effectiveness. Hopefully, this current paper will help health care providers be better able to respond to patient and parent queries about the varieties, possible mechanisms of action, and benefits of CAM therapies for childhood AD/HD. 

## 2. Botanicals 

### 2.1. Gingko Biloba and Ginseng

Abnormally low levels of dopaminergic activity in the prefrontal cortex have been implicated in childhood ADHD. In animal studies, ginkgo biloba increases dopaminergic activity [9]. In an open label, four-week trial, children receiving a combination of ginseng and gingko biloba were rated by parents as improved [10, 11]. Besides the limited treatment period and the absence of teacher ratings, the fact that some participants were taking concomitant stimulant medications limits drawing conclusions.

### 2.2. Pinus Pinaster Bark Extract

 Pycnogenol (Pyc), the trade name for French maritime pine bark extract, is believed to act as a vasodilator improving cerebral blood flow to brain regions involved in AD/HD [11]. Additionally, Pyc may regulate and modulate possibly elevated catecholamine levels among AD/HD children [12]. In a one-month RCT, children receiving Pyc demonstrated reductions in teacher-rated hyperactivity and inattention. After the washout period, symptoms returned to baseline. The paediatric patients also demonstrated improvement on fine motor coordination and concentration tasks [12]. However, a small clinical study of Pyc with AD/HD adults did not indicate that the herbal was superior to placebo [11, 13].

### 2.3. Hypericum Perforatum (St. John's Wort)

While probably being best known as a potential alternative treatment for major depressive disorder, hypercurium perforatum was the subject of one of the most rigorous CAM trials on AD/HD conducted to date [14]. In an eight-week RCT with pediatric patients, neither parent ratings of symptoms nor clinician ratings of global behavioral improvement significantly differed for St. John's Wort versus a placebo [14].

## 3. Minerals

 While findings are inconsistent, some investigators report abnormal levels of minerals among children with AD/HD including magnesium, copper, calcium, zinc, and iron deficiencies [15, 16]. A possible mechanism is that many minerals are cofactors for neurotransmitter synthesis, uptake, and breakdown [16]. Zinc, magnesium, iron, and lead have been the most commonly studied. 

### 3.1. Lead

Abnormally high lead levels have been associated with hyperactivity as well as cognitive deficits. While screening for lead is a common part of the initial evaluation of children with AD/HD symptoms, there is little evidence that mineral supplementation decreases lead absorption [11, 15, 17]. For example, iron and zinc supplements did not significantly reduce overactivity or inattention among lead-exposed children [18].

### 3.2. Iron

As a cofactor, iron is a modulator for synthesis of both norepinephrine and dopamine [11, 19]. Anemic children in several studies have demonstrated attentional deficits [11]. However, in the absence of anemia, iron supplementation in children with AD/HD has not been found to be associated with consistent improvement in parent or teacher behavior ratings [11]. 

### 3.3. Magnesium

Magnesium, as a cofactor for enzyme production, is involved in neurotransmitter synthesis and some studies have found magnesium deficiencies associated with AD/HD [19]. An RCT found that, compared with placebo, children with ADHD symptoms and magnesium deficiency receiving magnesium supplementation demonstrated significant improvement after six months of treatment. However, both groups were taking psychotropic medication [20].

### 3.4. Zinc

 Of the mineral supplements studied, the strongest evidence for clinical efficacy is for zinc. Low levels of zinc have been associated with deficits in several cognitive functions including information processing [11, 21]. Two studies of children with ADHD receiving zinc supplementation have found improvement based on rating scales [21, 22]. However, zinc was not used as the sole treatment in either study. There are suggestions that when added to conventional drug therapies such as methylphenidate, zinc may augment medication effects [11].

## 4. Essential Fatty Acids 

Compared with other nutrients, the role of EFAs in AD/HD and their therapeutic application has been subjected to several RCTs. Some investigations have found AD/HD to be associated with lower phospholipid levels and red cell membranes deficient in Omega-3s such as docosahexaenoic acid (DHA) [23]. While the exact mechanism for EFAs is not yet firmly established, it has been postulated that omega-3 fatty acids may act upon central nervous system cell membranes and phospholipid composition [24]. Increased omega-3 levels in cellular membranes, in turn, impact dopaminergic and serotonergic activity [24]. One study found improvement in parent-rated behavior and attentional tasks following 15 weeks of fish oil supplements [25] while another report indicated improved attention and global behavioral improvement for children receiving combined omega-3 and omega-6 supplements compared with olive oil [26]. A third RCT found significant sustained improvement for 30 weeks on cognitive tasks as well as on parent ratings with EFAs. However, teacher rated symptoms did not change significantly nor was there any additional benefit from adding a multivitamin supplement as augmentation [27]. A 30-week placebo-controlled trial of phosphatidylserine contained omega-3, followed by an open-label period, found a similar pattern of parent-reported benefits for restless-hyperactive symptoms in the absence of teacher-reported effects [28]. However, a subgroup of treated AD/HD children demonstrating particularly pronounced hyperactivity/impulsivity as well as oppositional behavior demonstrated a significant reduction in both parent- and teacher-rated restlessness and emotional lability [28].

Despite these positive findings, two other RCTs found that EFAs had minimal effect on AD/HD symptoms. Among children on stimulant therapy, four months of DHA supplementation were not associated with cognitive or behavioral benefits compared with placebo [29]. An additional study did not find any benefit from two months of EFA supplementation on either teacher or parent behavioral ratings or standardized cognitive tasks [30].

 In several RCTs reporting benefit from EFAs, the investigators employed a number of behavior ratings and cognitive tasks with few advantages for EFAs on most outcome measures. Another area of concern is that while biochemical measures indicated increases in omega-3 levels, a systematic association between EFA levels and behavioral outcomes has not been established [31]. In one study, omega-3 fatty acid supplements have been associated with a worsening of attention [27]. However, the frequent use of olive oil, a source of oleic acid which when converted to oleamide, has some psychoactive effects, as a placebo in this research may underestimate omeaga-3's benefits [25]. 

A meta-analysis found a modest effect (.31) for omega-3 FFA supplementation on AD/HD symptoms [24]. Finally, a Cochrane paper, while reporting tentative benefits for combined omega-3 and omega-6 supplements, concluded that there was little evidence that EFA's reduced AD/HD symptoms [32]. Currently, while demonstrating some promise as a complement to stimulant medication, the evidence base for EFAs is inconclusive.

## 5. Dietary Restrictions

### 5.1. Sugar Restriction

Many parents believe that excess sugar consumption causes hyperactivity. There have been two mechanisms suggested for this association. First, children with AD/HD may have a specific allergy to refined sugar [33]. A second view is that sugar ingestion influences cognition and behavior through a form of functional hypoglycemia. The latter hypothesis is supported by evidence of abnormal glucose metabolism among several groups of hyperactive children. A meta-analysis, conducted 15 years ago, concluded that there was no evidence that sugar ingestion caused hyperactivity or impacted cognitive performance [34]. However, a more recent paper was slightly more equivocal with 25% of studies finding some evidence of increased hyperactivity and inattention following sugar ingestion [33]. A qualification is that parental expectancies appear to play a role in this association. Mothers believing that sugar triggered symptoms were more critical and directive and more likely to rate their child as disruptive after being told that the child had ingested sugar rather than a placebo [34].

### 5.2. Food Additives

 In the 1970s, Feingold concluded that 50% of AD/HD children demonstrated particular sensitivity to dietary food colorings, preservatives, and natural salicylates and recommended diets free of these additives as treatment [35]. While early papers and a meta-analysis [36] concluded that the Feingold diet had little impact on AD/HD symptoms, recent research suggests that subgroups of children may be particularly sensitive to these substances. Additionally, nonclinical pediatric samples have demonstrated increases in hyperactivity after ingesting food dye and a preservative [37]. Among preschool children with AD/HD, a preservative- and artificial-flavoring-free diet was associated with decreased parent-rated hyperactivity. However, clinical evaluations did not indicate improvement [38]. Presently, it appears that subgroups of children may be particularly sensitive to artificial additives but this association is not specific to those with AD/HD [36–38].

### 5.3. Oligoantigenic Diet

 Several studies have suggested that food restriction followed by a systematic gradual reintroduction of offending foods may have some benefit in reducing hyperactive behavior [39–41]. While multiple “few foods” diets exist, a commonly employed restrictive diet was developed by Egger et al. [39] and includes a limited number of foods from each group including meats (lamb, turkey), carbohydrates (rice, potatoes), vegetables (carrots, cabbage), and fruits (apples, bananas). After the benefits of this usually well-tolerated diet are apparent, new foods are gradually reintroduced and in the case of AD/HD, behavior is carefully observed. While there are some suggestions that children with food allergies may show some behavioral improvement with the oligoantigenic approach, the clinical effects of this dietary intervention appear small—particularly when compared with methylphenidate [40]. Additionally, methodological issues such as small sample size, absence of blinding, and overreliance on parent ratings make the results difficult to interpret. It is likely that among children with both AD/HD and food allergies, dietary intervention may be a useful complementary therapy [40].

## 6. Homeopathy

Homeopathic medicine is based upon the concept of treating like with like. Any substance that may cause a specific illness is administered in reduced and/or diluted doses as a means of treating the condition. The treatment is often individualized based upon specific patients' symptoms and personality characteristics [41].

 A Cochrane paper located four studies meeting their criterion of random or quasirandom assignment [42]. The active treatments were either verum or mixtures of homeopathic substances including selenium and sodium phosphate. There was no evidence that homeopathic treatment was effective in reducing symptoms as reported on parent-completed behavioral rating scales or cognitive tasks [42]. Frei and colleagues, who conducted one of the trials, raised questions about the adequacy of evaluating outcomes based only on several months of treatment [43]. They noted that homeopathic treatments required an average of 6 1/2 months to reduce symptoms by 50%. Frei and colleagues also found that children treated with methylphenidate responded more slowly to homeopathic treatment even after stimulant medication had been discontinued [44].

## 7. Cognitive-Behavioral Interventions

### 7.1. Neurofeedback

ADHD has been associated with atypical patterns of cortical arousal. Quantitative EEG studies suggest that children with AD/HD frequently exhibit a pattern of cortical hypoarousal in brain regions associated with alertness, attention, and self-control. While less common, a smaller group of patients demonstrate hyperarousal with an increase in fast EEG frequencies over the same regions [45]. These abnormalities may reflect structural differences in neuroreceptor density as well as altered neurotransmitter activity for dopamine and norepinephrine with possible serotonin and acetylcholine involvement [45]. 

These atypical neurological findings are the basis of neurofeedback. While EEG monitoring occurs, children perform tasks while information about their neuroelectrical signals is translated into graphic displays, lights, or tones. In addition, patients may move cartoon-like video characters through cognitive-behavioral activity to maintain specific EEG patterns. The overall goal is for the patient to demonstrate a cortical activation pattern comparable to typical age peers for a 45-minute period [46]. Sessions are typically scheduled on a weekly or biweekly basis for 45 minutes to an hour. The average number of training sessions is 43 with a range from 34 to 50 [45–47]. 

Results of nonrandomized trials and a recent meta-analysis [48] indicated that children receiving neurofeedback demonstrated improved performance on attentional tasks such as the Test of Variable Attention (TOVA) as well as reduced parent-rated inattention and impulsivity [47–49]. A comparison of neurofeedback with another alternative therapy, attention skills training, found both treatments to be effective in reducing parent-rated AD/HD symptoms. However, neurofeedback was associated with greater reductions in both parent- and teacher-rated symptoms [50]. At least two studies have found neurofeedback to be equally effective as stimulant medication [51, 52]. Monastra found that those patients receiving neurofeedback appeared to maintain their cognitive-behavioral gains better when medication was withdrawn for a one-week washout. Additionally, among children taking stimulant medication, addition of neurofeedback was associated with dosage reduction [52].

While appearing promising, neurofeedback's evidence basis has been questioned [53]. To date, most neurofeedback studies are based on self (i.e., parent-)-selected samples and the majority rely on unblinded parent ratings and laboratory task performance as outcome measures. The absence of consistent teacher-rated improvement, together with the nonrepresentativeness of cognitive tasks to “real world” academic demands and the few posttreatment follow-up studies, raises concern about neurofeedback's generalizability. 

Nonspecific factors occurring during neurofeedback therapy such as the multiple months of regular therapist contact with children, repeated assessment of attentional skills, parent education, and behavior training make it difficult to conclude that EEG feedback is specifically responsible for the reported 60–75% improvement rates [52, 53]. In the recent meta-analysis, neurofeedback's comparative effectiveness was substantially less in the few randomized studies involving a “semiactive” control intervention [48] the time and cost (30 to 40 sessions at $75.00 to $150.00) may be prohibitive to many parents—particularly when not covered by insurance.

### 7.2. Cognitive Training

 Neuropsychologically, AD/HD is characterized by impaired executive functioning which includes planning, reasoning, and response inhibition [54]. A particularly important cognitive function underlying these skills is working memory—the ability to mentally “hold” information while performing operations such as calculations. Children and adults with AD/HD demonstrate pronounced working memory deficits. Laboratory studies indicate that stimulant medication improves performance on working memory tasks [55].

There are several commercially available computerized training programs designed to improve working memory and concentration among patients with AD/HD. *Cogmed,* a commercial program, includes visual-spatial and verbal tasks in which the patient briefly views a pattern or verbal material such as phonemes or letters on a screen and then applies it to a pattern on the subsequent screen. Training is often conducted in five 30- to 40-minute sessions for approximately five weeks. Once the child and their parents are familiar with the procedure, training sessions can be conducted on a home computer. Several clinical studies indicate significant pre-post training improvement on computerized tasks generalizing to novel cognitive tests [55–57]. In a study with a six-month followup after training, parents reported significantly improved attention was maintained. However, six-month posttreatment ratings by psychologists did not indicate clinically significant improvement in cognitive skills. In terms of effect size, the authors concluded that the benefits of training were comparable to methylphenidate [57].

 While memory training has been less well-studied than neurofeedback, the time commitment and cost are comparable. Commercial programs such as CogMed require that a specially trained “coach” be present physically or by phone during training. While promising, cognitive training is likely to be more effective for symptoms of inattention with significantly less benefit for hyperactivity. 

### 7.3. Yoga

 Research on yoga indicates that it impacts neurophysiological functions including oxygen consumption, lateralization patterns, and cognition—all of which often demonstrate atypical patterns in children with AD/HD [58]. While yoga is not a unitary set of techniques, a foundational procedure for AD/HD is respiratory training with a focus on rhythmic inhalation and exhalation that reduces sympathetic nervous system activity while providing an attentional focus [58]. In concert with breathing, postural exercises are conducted along with progressive relaxation. Finally, yoga may also include visual fixation on an object such as a candle flame or mental visualization of a word or shape. Two studies applying yoga to children with AD/HD have suggested that it may have some mild benefits that may additive with medication effects [58, 59]. There does appear to be a dose-response relationship with more sessions of yoga associated with greater improvement on teacher ratings of hyperactivity/impulsivity [58]. 

 Parents participating in yoga along with their children reported reductions in stress and improvement in managing their child. After treatment, parents rated their children as demonstrating improved self-esteem, and reduced behavioral problems [59].

### 7.4. Massage

 Several studies have examined the impact of therapeutic massage on adolescents with AD/HD [60, 61]. While the rationale for this therapy has not been well articulated, there is evidence that massage increases EEG patterns associated with attention as well as vagal ton [60, 62]. Increased vagal cardiac control may mediate increased motor inhibition [62, 63].

 A randomized controlled trial indicated that adolescents receiving weekly or biweekly massage therapy demonstrated improved mood as well as teacher rated classroom behavior [60, 61]. In addition, students with AD/HD were found to have significantly improved task focus—moving from being on task 47% of the time to 75% at the end of ten consecutive school days of 15-minute massage sessions [60]. The length of the intervention—a total of 10 days to four weeks—is unlikely to have enduring benefits after regular massage has ended. Additionally, in the few studies conducted, medication status was unclear.

### 7.5. Meditation

 Mindfulness meditation involves training people to be observers of their ongoing thoughts and feelings without attempting to change these internal experiences. Several studies of mindfulness meditation in adults have suggested that it may have beneficial effects on cognitive activities such as shifting set and possibly, in improving working memory [47]. There are relatively few studies on meditation techniques applied to childhood AD/HD and the reports are based on small sample sizes. Results to date have been mixed with one study indicating improvement in parent ratings of impulsivity and improved performance on an attentional measure and another indicating improved classroom behavior in the absence of parent reported improvement [64, 65].

### 7.6. Green Space

Green Space is the term used to describe a natural green setting including trees, and grass. Green space exposure as a form of treatment is based on Attention Restoration Theory (ART). ART posits two forms of attention: involuntary and voluntary. In this theory, attention deficit stems from overtaxed or fatigued voluntary attention. When voluntary attention demands are greater, sustaining attention becomes more effortful and ineffective. As with rest or sleep, activities that draw upon involuntary attention permit voluntary attention to recover. ART adherents believe that different types of environment have differential effects on attention [66]. Those environments, such as the classroom requiring more effortful forms of attention, are fatiguing. In contrast, outdoor environments with green space are gently absorbing and draw upon involuntary attention while restoring voluntary attention [66].

In a survey, parents of children with ADHD reported greater symptom improvement after children participated in activities in “natural" settings versus indoor or artificially built outdoor settings such as cement playgrounds [67]. While direct pre-post green space exposure studies are limited, a recent study found that children with ADHD performed better on a verbal task sensitive to concentration after taking a walk in a park versus a residential or downtown setting [68]. Effect sizes were comparable to those associated with methylphenidate treatment [68]. While interesting, there are little data to indicate the duration of green space exposure required or the duration of improved cognitive functioning after exposure.

## 8. Conclusion 

 To date, complementary and alternative therapies for childhood attention deficit hyperactivity disorder have not been consistently supported. Relatively few studies include randomized controlled placebo trials. Conducting randomized controlled trials is particularly challenging in complementary and alternative medicine since an important therapeutic factor appears to be participants' belief in CAM efficacy as well as their relationship with the provider [8]. Additionally, interventions such as neurofeedback and memory training, requiring multiple months of individualized sessions, may be beneficial to patients and their parents because of the nonspecific factor of individualized attention from a mental health provider. Because of self-selection and expectancy effects as well as the current unknown efficacy of CAM versus the established effectiveness of stimulant medication, it has been difficult to conduct true randomized CAM trials for AD/HD. In addition, many of the trials papered had particularly large attrition rates. 

Of the therapies papered, neurofeedback appears to have the strongest evidence basis. This treatment warrants further investigation. It is noted, however, that most of the published trials of this treatment, while including some of the limitations noted immediately above, are also authored by those with a strong allegiance to this therapy. Of the nutritional supplements papered, omega-3 oils and possibly zinc [69] may also have some additive benefit in conjunction with stimulant pharmacotherapy.

The possible negative effects of nearly all of the treatments paper are negligible. Given that very few of these treatments have been tested in head to head trials with established therapies such as methylphenidate, it is difficult to make definitive conclusions regarding possible clinical efficacy. Nonetheless, the consistent finding that up to 30% of children with AD/HD do not respond to stimulant medication and the physical side effects of pharmacotherapy suggests that there is a definite role for efficacious alternative and complementary therapies. However, investigations to date suggest that as the rigor of research design increases, the likelihood of finding a positive effect for alternative therapies for AD/HD decreases [8]. Therapies such a neurofeedback, omega-3s, or zinc supplements, which may be beneficial, may demonstrate optimal benefit when complementing established treatments including stimulant medication.
